# Exploring structural violence in the context of disability and poverty in Zimbabwe

**DOI:** 10.4102/ajod.v6i0.274

**Published:** 2017-04-18

**Authors:** Jennifer T. Muderedzi, Arne H. Eide, Stine H. Braathen, Babill Stray-Pedersen

**Affiliations:** 1 Institute of Clinical Medicine, University of Oslo, Norway; 2 SINTEF Technology and Society, Norway; 3 Division of Women-Rikshospitalet; Oslo University Hospital, Oslo, Norway

## Abstract

**Background:**

While it is widely assumed that disability, poverty and health are closely linked, research falls short of fully understanding the link. One approach to analysing the links between disability and poverty is through the concept of structural violence, referring to social structures that contribute to the impoverishment of individuals or communities. These structures can be political, ecological, legal and economic, among others.

**Objective:**

To explore structural violence and how it affects families of children with cerebral palsy among the Tonga ethnic group living in poor rural communities of Binga in Zimbabwe.

**Method:**

This is a longitudinal, qualitative and ethnographic study. Data were collected over a period of eight years from 2005 to 2013. Data collection techniques were in-depth interviews, participant observation and focus group discussions. Purposive sampling was used to recruit 53 informants.

**Results:**

Structural violence was noted through four themes: internal displacement and development, food and politics, water and sanitation, and social services. Poverty was noted in the form of unemployment, lack of education, healthcare, food and shelter. The concept of structural violence inflicted social suffering on the informants. Politics played a major role in activities such as food withdrawal, lack of water, development and allocation of local resources to ‘the people of the city’, leaving the informants struggling with care.

**Conclusion:**

Political and economic forces have structured risks and created a situation of extreme human suffering. The capabilities approach brings out the challenges associated with cerebral palsy in the context of development challenges.

## Introduction

The concomitant relationship between poverty and disability is well established in the literature (Ansell 2005; Emmett & Alant 2006; Palmer 2011; Rousso 2003), and we are beginning to understand the complexities of the relationship. However, as Meekosha (2011) and Grech (2009) argue, literature in the field is often dominated by ‘northern’ perspectives that fail to understand the challenges associated with disability in the context of wider development challenges, and evidence is often anecdotal rather than evidence-based (Groce et al. 2011). These links are more complex and nuanced than currently assumed (Barron & Ncube 2010; Groce et al. 2011).

What people can positively achieve is influenced by economic opportunities, political liberties, social powers, and the enabling conditions of good health, basic education, and the encouragement and cultivation of initiatives. The institutional arrangements for these opportunities are also influenced by the exercise of people’s freedoms, through the liberty to participate (Sen 1999:5). Graham, Moodley and Selipsky (2012) state that the reality for many people, and particularly for people living with disabilities, is that their capabilities are very much constrained, thus limiting their opportunities to achieve particular desirable functionings.

Different disabilities represent different impairments and challenges and it can be fruitful to differentiate and study disabilities and impairment categories separately to get more in-depth information. Cerebral palsy is the most frequent cause of physical disability in children, occurring in approximately 2 to 2.5 per 1000 live births (WHO 2001) and 1.5 to 5.6 cases per 1000 live births in the developing world, with a higher incidence in boys than in girls (1.33:1) (Stanley, Blair & Alberman 2000). Burton’s (2015) study in Botswana states a possible prevalence of up to 10 cases per 1000 births, resulting in a lot of children who are not able to reach their potential.

Common characteristics of cerebral palsy are loss of motor function, complications of epilepsy, vision, hearing, intellectual, stunting, speech and feeding problems, resulting in chronic conditions that need a lot of care. Apart from physical problems such as muscle weakness, stiffness and clumsiness, people with cerebral palsy are also four times more likely than their peers to experience emotional and behavioural problems (Sigurdardottir et al. 2010). It is therefore not surprising that cerebral palsy can have profound effects on the physical, social and emotional health and well-being of children and their carers (Glenn et al. 2009). Families of children affected by cerebral palsy were chosen for this particular study because of a high incidence as reported by village health workers in the study area. Cerebral palsy is also particularly interesting because of the multiple impairments and challenges often associated with the condition.

The purpose of this article is to explore how structural mechanisms play out and negatively affect families of children with cerebral palsy among poverty-stricken Batonga in Zimbabwe.

## Background

Malnutrition, disease, less access to safe drinking water, inadequate sanitation, poor healthcare and a lack of rehabilitation services are all causes of disability in the developing world (Qayyum, Lasi and Rafique 2013; Saetermoe et al. 2004). Regarding children with significant physical and sensory disabilities, it is recognised that with the right supports these children can have a better chance to live independent lives and contribute economically and socially to society. However, children with disabilities are often denied the right to basic education and healthcare, losing their chance to become independent (Global Partnership for Children 2012; Zinkin & McConachie 1995). Having a child with disability thus often leads to households experiencing multiple dimensions of poverty, causing them to go deeper into poverty and making it likely that they will never escape impoverished living (Park, Turnbull & Turnbull 2002).

The Convention on the Rights of the Child (CRC; UN 1989) and the Convention on the Rights of People with Disabilities (CRPD; UN 2006) state that children with disabilities have the same rights as other children to healthcare, nutrition, education, social inclusion and protection from violence, abuse and neglect. Ensuring access to appropriate support, such as early childhood intervention and education, can fulfil the rights of children with disabilities, promoting rich and fulfilling childhoods and preparing them for full and meaningful participation in adulthood (Simeonsson 2000). Barriers such as inadequate services and lack of accessible environments create more problems for children with disabilities and their parents. If children with developmental delays or disabilities and their families are not provided with timely and appropriate early intervention, support and protection, their difficulties can become more severe – often leading to lifetime consequences, increased poverty and profound exclusion (United Nations Fund, World Health Organization & The World Bank 2012).

Many children with disabilities in Zimbabwe are failing to realise their full potential as they struggle to achieve basic human rights such as access to education and healthcare services (Marongwe & Mate 2007; Muderedzi 2006; UNICEF-Zimbabwe 2013). Most research in Zimbabwe has been carried out in typical communities where disability and poverty have been noted to be intertwined (Marongwe & Mate 2007; Van der Mark & Verrest 2014). Research on caregivers of children with disabilities in Zimbabwe remains sparse, especially in remote rural areas where cerebral palsy is a common disability in children because of lack of health services as well as cultural and religious practices. Most villagers’ poor economic situation, lack of road infrastructure, culture or religion restrict them from accessing health centres leading to giving birth at home and thus increasing risk of complications that can result in a cerebral palsied child (Eide & Ingstad 2011; Muderedzi 2006).

Qualitative studies, for example, Muderedzi and Ingstad (2011) using the ‘Ingstad and Sommerschilds’ (1983) model for coping with disability, have gone further to show the relevance of cultural, political and structural phenomena in relation to disability and poverty. The model, though focusing on the individual, integrates explanatory variables outside of the individual.

Because of a small but growing evidence base indicating that there are substantial links between disability, poverty and health (Groce et al. 2011), this article aims to bridge the gap in knowledge by exploring structural violence, the harm it causes and its effects on households of children with disabilities in poverty-stricken communities among the Tonga in the north-west of Zimbabwe.

### Structural violence

Farmer et al. (2006) define ‘structural violence’ as one way of describing social arrangements that put individuals and populations in harm’s way. The arrangements are structural because they are embedded in the political and economic organisation of our social world; they are violent because they cause injury to people. Structural violence addresses poverty and injustice and tries to understand the real dynamics of suffering and poverty, and understand political economy and how power works (Farmer 2004). The concept was developed in relation to social suffering, power and inequality. It draws attention away from individual responsibility and increases understanding of how social experiences are being shaped through structural processes of historical, political and cultural context. Tonga history of decades of chronic poverty and suffering called for the concept of structural violence to illustrate the links between disability and poverty.

### The Tonga

The Tonga is an ethnic minority group in Zimbabwe, living in Binga district, on the north-west borders of Zambia and Zimbabwe. The forced displacement from the Zambezi valley in 1957 for the construction of Lake Kariba caused a disruption in their socioeconomic and cultural environment as they were relocated to higher, dry country marred by low erratic rainfall, poor rocky and sandy soils, tsetse fly and mosquito infestation and marauding wild animals. Out of the 59 districts in Zimbabwe, Binga has been the most neglected as it lacks essential services such as health, education, communication and agricultural development (Mashingaidze 2013).

Swift and De Graaf (1994) state that the Tonga lived in a terrible state with 60%–80% child mortality because of lack of vaccinations, malaria, diarrhoea, tuberculosis, leprosy and bilharzia in the adult population. They suffer from acute food and water shortages and are heavily reliant on national and international food aid, resulting in them being labelled ‘donor dependent’ and ‘lazy’ by people who do not know their histories and biographies. The situation still prevails with new infections such as HIV/AIDS and communicable diseases. Matabeleland North province where Binga district is situated has the highest poverty rate in the country with 70% of its inhabitants classified as poor or extremely poor (Basilwizi Trust 2004; Rural Poverty Portal 2015; Zimbabwe National Statistics Agency [ZIMSTAT] and ICF International 2012).

## Materials and methods

The study was longitudinal, qualitative and ethnographic in nature (Sandelowski & Barroso 2003; Silverman 2013). The researcher returned to explore changes that occurred over time and the processes associated with these changes (Farrall 2006). Data collection techniques included 53 in-depth interviews, 20 participant observations and 10 focus group discussions conducted in the local language Chitonga. The first cohort (2005) consisted of 30 infants (birth–5 years of age) of which 23 died during the first two years of the study from malnutrition, diarrhoea, malaria, meningitis and other infections. The second cohort (2012) consisted of 7 from the first cohort and 23 new recruits (8–13 years old). A pilot study was carried out before the main study.

Purposive sampling of 53 families was chosen because it illustrated some features or process in which we were interested (Silverman 2013). Caregivers were selected purposively based on the groups which the research question and theory addressed. Informants were chosen on parameters such as Tonga caregivers, children with cerebral palsy and age groups (birth–5 years; 8–13 years). Caregivers were parents, grandparents, aunties and siblings who were selected to represent these different categories. Forty were selected from the research assistant’s community and hospital list and 13 were through ‘snowball’ sampling using social networks of initial informants. Three were child-headed families. To improve the likelihood of accuracy and objectivity, the project used a triangulation of three methods of in-depth interviews, participant observation and focus group discussions, as well as field notes and secondary data (documents).

In-depth semi-structured interviews were conducted among all the 53 families to elicit information such as their experiences of life before and after having a disabled child, beliefs and attitudes towards disability, effects of caring for a disabled child and factors influencing the caring of the child such as social, economic or political. The aim was to encourage informants to speak personally and at length about their lives as caregivers. Participant observation was chosen for its part as a process of enculturation (Schensul, Schensul & LeCompte 1999) where the researcher absorbs the big picture and some details leading to an understanding of people’s daily lives, structure and events, social structure, expectations and values. It also allows for the juxtaposition of what people say they do and what they are observed to do. Twenty informants were selected to participate. Focus group discussions were used to assist informants to further explore and clarify their points of view.

During in-depth interviews, the researchers stayed at clinics. Participant observation was carried out during three days stay at an informant’s homestead with the researchers immersed in most activities of daily living (ADL) such as fetching water and wild fruits, teaching handling techniques of the disabled child and community gatherings so as to gain more depth and insight into informants’ lives. This also allowed the researcher to meet the extended family and neighbours especially in the evenings where disability songs, stories and life in the Zambezi valley were narrated. It is also important to note that family experiences of living with both disabled and non-disabled children were noted as the model used for the study (Model for Coping with Disability; Ingstad and Sommerschild 1983) made the household unit the main focus of investigation including the community.

### Data analysis

Data were translated into English and transcribed verbatim. The data were labelled through open coding, using conceptual categories to develop the codes (Silverman 2013). The codes were derived both from the literature and the actual data (Corbin & Strauss 1990, 2008). This was read several times and factors that were associated with structural violence were identified. Sen’s (1992) capabilities approach was used to demonstrate ways in which both disability and poverty compound one another to limit the capabilities of the informants.

A ‘thank you’ token of $5 was given to each family during in-depth interviews. Families who were part of the participant observation received $10 worth of groceries as they were hosting the researchers for three days. Caregivers were given advice on child handling techniques and child care information. Children with disabilities were shown how to carry out ADL such as washing, dressing, feeding and mobility. Referrals to specialists and health centres were not feasible because of caregivers’ economic hardships.

## Results

Using the theoretical framework of structural violence in the context of disability, factors that affected families of children with disabilities and seen to cause social suffering were identified as follows: internal displacement and development, food and elections, water and sanitation, and social services.

### Internal displacement and development

The ‘river’ Tonga (generation evicted from the Zambezi valley in 1957) stated how the forced displacement from the ecologically rich river plains to dislocated communities had disrupted their socioeconomic and cultural environment. The Tonga are forced to share the district with wildlife that escapes from the nearby game reserve. Elephants are known to attack and kill villagers as well as destroy their harvests. Drought conditions account for hunger and famine episodes affecting both humans and livestock with lack of food and water. The floods destroyed their ancestral burial grounds and effectively disrupted their whole way of life. They were completely cut off from family and friends living across the river in Zambia. Most of them reported of not being able to take part in ceremonies to commemorate their ancestors for protection and other misfortunes stating that their gods never helped them again, stating:

‘More attention was paid to the animals and rescuing them than us. … Some people were attacked by wild animals in the open spaces. … We can no longer perform traditional ceremonies in honour of our ancestral spirits (*mizimu*) – the river connected us to our relatives and friends (*bamutala*) on the other side of the river.’ (an Old grandfather, in his 80s, married with five wives)

‘We had a good life. The soil was fertile and we had more water than we needed. We grew corn, sweet potatoes, bananas, vegetables and other crops. We had plenty of fish and wild animals for food. Life in Kasambabezi (Zambezi valley) was good.’ (Old grandmother, partially blind, in her 80s)

Drought conditions accounted for hunger and famine episodes affecting both humans and livestock with lack of food and water. Most young men stated:

‘The Zambezi river is our only salvation … there should be unhindered access to it, but the problem is that the Tonga are virtually barred from fishing from it because of prohibitive levies and this is driving poverty levels up. Instead the river is now benefitting fishers and traders from the cities and towns who have the money to pay levies.’ (Young, unemployed, married man in his 30s, with three children)

Displacement resulted in the loss of unity and togetherness leading to lack of much-needed help and support from the extended family, especially young families with a disabled child who needed constant healthcare support with traditional medicines. Reports of negative impacts on social functioning because of long distances and wild animal attacks were reported as causing anguish especially to informants who had to carry the older disabled child on their backs as well as those residing in remote mountain areas. Social isolation was reported by many of the informants. A mother in her 30s with four other children stated:

‘He is always sick. I have to stay at home most of the time when my husband visits our relatives and friends. … My lower back hurts each time I carry him for long distances, so visiting relatives in not possible. … I sometimes feel depressed and find it difficult to cope, if only my sisters lived nearby they could help us. … I am always tired but it’s ok. He is my child.’ (A Mother, in her 30s, with four other children)

The ‘river Tonga’ women lamented their lost fertile land that enabled them to produce two harvests per year resulting in plenty of food and no famines.

The Tonga reported of the authorities not allowing them to participate in decisions relating to the evictions and the resettlement. Development projects such as fisheries, tourism, wildlife, timber resources, minerals, electricity generation and employment, which could have improved their lives through good nutrition, education, healthcare, electricity, modern shelter with water and sanitation facilities, were not availed resulting in chronic poverty. The dam was not effective for the displaced did not enjoy the benefits. They reported of not receiving any compensation to date. The country’s socioeconomic development has eluded the district leading to subsistence activities of farming, collection of forest produce (famine foods) and non-governmental organisation (NGO) food aid. Poverty, unemployment and lack of road infrastructure and social services resulted in increased disability incidences, disease or death because of lack of access to healthcare and government health programmes. Some of the informants stated:

‘If one of us, falls sick, they will die. … my wife and baby died during labour for lack of transport and road infrastructure to the nearest health centre (20 kilometres away) … My son cannot walk, he is not able to go to school and will never have a job.’ (Man in his 50s, married, with two wives and nine children)

‘He is not able to sit unaided, crawl or speak. He has no head control and has to be carried all the time. … He cries a lot during the day and worse still at night. I cannot sleep most nights. … He drools all the time, cannot feed properly, and it takes a long time to feed him. I wish I had proper food to feed him. Look, he is small for his age.’ (Married woman, in her 20s, with two other children)

People in general and more so disabled children, found the narrow winding paths extremely difficult to negotiate their way to school, healthcare and visits to friends and the extended family. Most disabled children not attending school stated:

‘I cannot run away from elephants when they attack children from school. … We have no food or water to take to school. … The school is very far. … My parents cannot afford the school fees. … There are no bridges on the rivers for us to cross safely to school.’ (100 year-old, boy, with difficulty in walking)

The four disabled children who were attending school in the district were illiterate because of lack of special needs schools and teachers. As a result, there was no child who managed to finish primary school education. Exclusion and discrimination deprived the disabled child from attaining an education and future employment. Generally, Binga lacks educational facilities for all.

The extent of their deprivation and suffering was noted in their new location, poor livelihoods and few resources. The government seemed to care more for the wildlife in the game reserve by providing water holes and fencing although some were breaking down leading to wildlife invading the communities for food and water.

### Food and politics

When asked for urgent intervention on food shortages, one government minister stated:

‘Most of these NGOs play politics with food and they might as well use the food handouts to influence our people to vote for the imperial lapdogs, the MDC. We are busy with Senate elections and after that we will look at the situation. But it should not be lost that we have the capacity to feed our own people.’ (Zimbabwe Print Media, 14–20 October 2005)

in contrast, informants were stating:

‘Quelea birds destroy our fields every year. … We were not threatened by elephants before, but now they are everywhere. We no longer bang empty tins to scare them away lest we anger them, but watch as they descend on our fields and graze on our crops … with drought persisting, the number of deaths may increase still more as elephants and humans compete for resources.’ (40-year-old married man, with 10 children and 2 wives)

‘We now have one meal a day. … We no longer have the staple food of maize or sorghum. … We are surviving on wild leaf okra without the starch. … We last had something to eat two days ago. … We have no proper food to feed the disabled child. He cannot chew the wild fruits or dried game meat.’ (A Mother, in her 30s, married with four other children)

The study showed an excessively high infant mortality (23 out of 30 from the first cohort and 7 from the second cohort) because of disease and chronic malnutrition. Only three informants living near the fisheries, an irrigation scheme and the main road were noted to have less food shortages compared with the rest of the informants.

The researcher witnessed government’s politicisation of food aid distribution as well as the barring of food aid by NGOs in the district resulting in chronic food shortages leading the informants to survive on ‘famine foods’ such as wild fruits, roots and leaves. Government’s withdrawal of nutritious porridge for the under-five infants was noted mainly during election times. Delivery of the porridge from the district hospital to central points would be stopped and mothers of sick children were stopped taking porridge home after discharge from health centres. Several visits to the district hospital showed distressed women (two informants included) stating that the children had improved during the hospital stay but were going to die at home because of lack of food. ‘Baby food’ such as mash and easy to swallow foods were lacking, leading to frail children with or without disabilities not being able to eat properly and succumbing to malnutrition, weight loss, stunting, developmental delays and death. Wasting, delayed milestones and stunting were noted in 37 of the children. Most of the informants’ sentiments were to see an improvement in their children’s health, stating:

‘I need food to improve the child’s health status so that he is able to sit and walk like other children.’ (16-year-old, married woman, with two children)

Child mortality was witnessed with affected informants stating:

‘In the end, my child died from hunger. There was no proper food to feed her.’ (20-year-old woman, with four other children)

A chronic food shortage among the informants was noted to perpetuate the spread of HIV/AIDS. The informants, especially women, reported of having undergone several HIV tests anticipating a positive result in order to access food aid from the few NGOs that carried out Voluntary Counselling and Testing (VCT) programmes in the district.

### Water and sanitation: ‘Go, the water will follow’

The above was a statement by district administrators during the evictions when the ‘river’ Tonga asked how they were going to survive on the dry mainland, maintaining that they were still waiting for their water to follow them, stating:

‘If there had been no dam, we would not be suffering like this. … Our land was taken away so that the people of the city can have electricity. … Now the government is planning to send water to the people of the city 450 kilometres away. … We want our water to follow us.’ (Old man, in his 70s, living with a family of four wives and grandchildren)

Informants stated how dehumanising this was to them as a people. Drinking water was sourced from open ponds, wells and dried-up river beds (competing with animals), with only a few having access to boreholes. Women had to walk up to 20 kilometres to fetch water twice a day in the scorching heat. This was a huge constraint and anguish to informants who had to carry the disabled child on their backs during such strenuous household chores resulting in reports of backache and chest pains. They reported of not being able to leave the child at home because of the child’s problems such as athetoid palsy (flaccidity) or repetitive behaviours (head banging or crying) that would make it difficult for siblings or co-wives to cope with.

Lack of piped water, boreholes, asbestos or tin roofs resulted in the consumption of contaminated water especially during the rainy season for lack of sanitation. Only one family had sanitation in the form of a dug-out pit while the rest practised open defecation. Cholera outbreaks, infections and diarrhoea mainly affected the under five-year-olds, the sick and the elderly. Bathing was a luxury especially during the dry season when water was scarce. It was common to hear female informants stating:

‘We haven’t had soap for a very long time and have forgotten what it looks like (laughs). … These days we only get enough water for cooking and drinking and not for laundry or bathing.’ (Woman, in her 20s, married with three children)

Hygienic processes such as washing of children’s soiled clothes were compromised, leading to more infant infections and anguish to the informants who could not access healthcare for their children. Some villages were void of fresh water, relying on salty unpalatable water instead. The consumption of salty water was noted with adverse effects of yellow corroded teeth among the children. Vegetable gardens were not possible since the water was not conducive to plant life.

### Health services

Out of the 53 children, 8 were born at a health centre, 14 were immunised, 10 attended health centres at least once for treatment, 4 received rehabilitation services, and all 53 needed therapy and assistive devices which were out of reach for most of them. Lack of access to health services because of financial constraints, lack of health centres, medical personnel, medicines, long distances and lack of public transport were constraints that affected the whole district. Informants reported emotional and practical suffering in dealing with multiple healthcare-seeking procedures with the disabled child compared with when they had non-disabled children, resulting in a lot of anguish and disruption in the family. Apart from birth, disabilities were also acquired later in childhood through malnutrition, disease, HIV/AIDS, lack of vaccines and accidents.

The following narration shows the anguish caused by poverty, lack of and access to social services and development to the district:

‘My child was not like other children his age between 5–6 months of age. He had no head control and could not sit up. My fears were confirmed when at 10 months he could not sit up. The first port of call was the traditional healer’s place but the family had no money and so we went to the prophet at the church of Zion who stated that the child was disabled and would never walk. My brother who works in Bulawayo then sent some money and we took the child to Binga rehabilitation department where I was shown some exercises to do and not the cause of the disability. After this we visited the traditional healer who confirmed that the child was bewitched. The problems with the child are that he is always sick and needs medication that we cannot afford and that he cries all the time and so stays on my back all the time. My biggest problem is the fact that he cannot sit or crawl like other children. I am now worried about the coming rainy season whereby I should be working in the fields with the rest of the family. Meanwhile I cannot carry out “piece jobs” for other families such as cutting thatching grass or take part in community activities.’ (Young mother, in her 20s, married with two other children)

Having a disabled child necessitated visits to the traditional healer to find out ‘why’ it happened and later for treatments. The Tonga believed causes of disability to be ancestors’ sorrow or witchcraft. If not, the ‘will of God or it just happened’ resulting in not putting blame on the family or the child. The child was referred to as *mwana murema* meaning a disabled child and taken care of as well as its siblings.

Tonga women referred to childbirth as:

‘A process that could go either way; right or wrong and depended on your luck.’ (an Old lady, in her 60s, who had lost three children during childbirth)

Maternal and high infant mortality during labour created anguish for the families with many wishing they could access modern healthcare. Excessive bleeding and complications during and after giving birth were reported as major causes with malaria, malnutrition, diarrhoea, infections and other diseases.

Poverty resulted in many of the informants selling mosquito nets received from government or NGOs in order to buy food or medication for the sick disabled child when in actual fact the child needed malaria prevention.

Reports of seizures, lack of ADL and emotional and behavioural problems were very common among the disabled children.

‘He has seizures most of the time and now he seems to have lost his sight and hearing – he does not smile or turn his heard towards sounds.’ (Grandmother, in her 60s, taking care of a four- year-old grandchild)

‘He cannot do anything for himself. I have to wash, dress, feed and carry him on my back or lay him down.’ (Mother of a three- year-old boy, with no muscle control)

Parents of a four-year-old boy and five-year-old girl with emotional and behavioural problems stated:

‘We have to tie his arm to a tree at the homestead so that he does not escape from home. He has a tendency to wonder in the forest and we fear he might drown or fall over a cliff. He is young so he is not able to untie himself – we untie him when his father and brothers are at home so as to run after him when he escapes.’ (Married mother of a four-year-old boy, in her 40s)

‘Our daughter uses foul language when we have visitors. … It is very embarrassing and stressful for the family.’ (Married woman, in her 30s, with five other children)

Lack of clothes was noted, with informants wearing ‘hand- me-downs’ from family members in nearby towns or well-wishers. Suspected HIV-positive disabled children suffered a double blow of infections and problems relating to disability such as cognitive and motor delays. HIV-positive informants and their disabled children were noted to be struggling to survive without anti-retroviral treatment with children who could have been HIV negative if prevention of mother-to-child transmission (PMTCT) services were available. VCT services have yet to reach the majority of the informants while most parts of the country are receiving the services.

It was also noted that for those receiving the services, the Information, Education and Communication (IEC) materials were not in the local language, disadvantaging the communities. Lack of appropriate services for disabled children such as community-based rehabilitation (CBR) was a barrier to independence, limiting ADL skills resulting in total dependency on caregivers. This was a major issue especially among adolescents who needed privacy with activities such as bathing or toileting.

## Ethical consideration

Ethical approval was obtained from the Medical Research Council of Zimbabwe (MRCZ – Ref. MRCZ/B/286) and the Regional Medical Ethics Committee (REK – case 2015/397) in Norway. The aim of the study was explained to the informants who gave their consent by signing a consent form or putting an ‘X’ figure as signature. The families were identified by numbers to maintain anonymity and confidentiality. The researcher and first author of this article is Zimbabwean with a fairly good command of Chitonga. The informants were open minded, hospitable, eager to participate and willing to tell their stories. Furthermore, the research assistant was a Tonga rehabilitation technician with over 30 years’ experience working in the district. She helped with explanations of Tonga culture, and facilitated negotiations for entry to the district through community chiefs and headmen, the district administrator and access to the informants.

## Discussion

According to the World Commission on Dams (2000), the resettlement programme was removed from the main Kariba project, a deliberate act to disadvantage the Tonga. They were not allowed to participate in decisions involving the planning of their resettlement and efforts were not made to minimise the negative impact of the displacement. The potential use of the Kariba water for irrigation was not investigated as part of the project. Displacement resulted in loss of cultural identity when materials of cultural values and shrines were left behind, loss of livelihoods as well as unprotected settlements that exposed them to wild animal attacks. Healthcare and education were virtually absent, communication and transport were non-existent, and travel was mainly by foot along footpaths which exposed them to wild animal attacks. Lack of equitable distribution of benefits from the project such as improved living standards, water, electricity, social services and employment showed how Kariba has failed the test of development effectiveness.

Binga appears to have missed out on the massive development programmes implemented in the early years of independence by the government and NGOs. The country’s independence (1980) did not usher in much improvement to the district as in the rest of the country. Political violence in the form of food aid withdrawal and lack of development has resulted in a life of abject misery and extreme human suffering. The forced relocation had a dramatic effect on their lives, the consequences of which are still felt today (Conyers 2002; Cumanzala 2002). Their quality of life has not changed for the better pre- or post-independent Zimbabwe, stating that the ‘past is the present’. Structural violence was the root cause of their problems which were not found in personal responsibility but in displacement of communities by a dam planned by powerful actors, which has to date created a situation of extreme human suffering.

Cerebral palsy was seen to create a lot of suffering for the informants and their disabled children. Insomnia, anxiety, stress, tiredness and depression were reported and noted to affect some of their daily activities. Reports of anguish, stress and anger of seeing their disabled child with no education, a future without a job meant perpetual poverty for both the child and family. Broadly, the challenges included psychological ones owing to caregiver demands and uncertainties and physical health challenges that emanated from excessive stress and through constantly assisting their children in ADLs as also noted by Sajedi et al. (2010). Lack of financial help and isolation led to reports of depression, which affected crucial activities such as fieldwork that would lead to less yields. Lack of mobility led to food poverty because of loss of manpower where the child would have contributed as part of the household by working in the fields as well as fetching wild fruits for family consumption during the famine months. Traditional healer treatments in the hope of improving the child’s disability resulted in economic challenges with some reporting being in debt. Cerebral palsy, with its numerous comorbidities, was seen to maintain the disability poverty vicious cycle because of the problems that it created affecting the whole family.

The study results show structural violence in the form of exclusion and discrimination as the current evidence base for the link between disability, poverty and health. Cerebral palsy was noted to result in structural power relations and the production of inequalities. Structural violence took away the informants’ capability to pursue and achieve well-being of their families and their disabled children. The government’s deliberate oversight of development in the district has led to lack of opportunities for the Tonga. They were noted to lack political liberties and social powers that other districts in the country enjoyed. Their suffering was caused by lack of water and electricity from the Zambezi River, government food aid withdrawal, high unemployment, lack of social services, poor road infrastructure and a nomadic life style – factors that the government through goodwill could have eliminated decades ago and enabled them to achieve well-being (Colson 1971; Currey 2009; Tremmel & the River Tonga People 1994).

Structural violence was seen to create social suffering among the disabled children as well as the carers who had to cope in an environment that lacked social services and social support. Characteristics of cerebral palsy such as abnormal brain development caused by bacterial or viral infections prior to birth, labour, delivery complications and disease complications such as meningitis and maternal hypertension could be minimised, as well as cerebral management with drug treatment, orthopaedic surgery, orthotics and different therapies with accessible and affordable healthcare, minimising the disability incidence. Mobility, a major activity among these communities for survival, was denied the disabled child creating major challenges to family care. A vicious cycle of poverty because of structural violence was amplifying the negative consequences of disability.

The late 1980s ushered in Tonga mediating institutions such as the Binga Development Association (BDA), the Binga Catholic Commission for Peace and Justice (BCCPJ) and Basilwizi Trust which sought to empower the marginalised people of the Zambezi valley by advocating for stronger government commitments to the development of the district’s infrastructure such as schools, hospitals and irrigation schemes. Unfortunately for the Tonga, government structures infiltrated the BDA and BCCPJ structures and destroyed them in the late 1990s leaving Basilwizi Trust to continue the fight. The continued occupancy of the Binga Tonga of an environment with little support for food production as well as lack of government support shows their resilience against ecological threats and social structures. The capabilities approach in this case has been used as a lens for understanding disability as a development concern (Graham et al. 2012).

The capability approach (Sen 2009:258) argues that disabled people face not only impairment of income-earning ability (earning handicap) but also the challenge of converting income or resources to achieve a valued life (conversion handicap). Structural violence was noted to have destroyed the informants’ income-earning ability by lack of development to the district resulting in chronic poverty that affected their disabled children’s future. Both the earning and the conversion handicaps were missing among the informants violating the social model of disability and its human rights approach for the disabled child. Their situation was a lack of resources to harness opportunities. Participation contributes to the quality of people’s lives, and the ability to do something for other members of the society is an elementary and valued freedom (Dreze & Sen 1995). Lack of participation for the disabled child and parents in different programmes such as education, peers, family and community at large was noted to result in lack of quality of life, helplessness and a bleak future. The children’s situation as they grew into adulthood was noted with the notion of ‘disability is inability’, a notion that the disability world is moving away from. Munsaka and Charnley’s (2013) study among adult Tonga of Binga with disabilities posted the same sentiments such as lack of participation, accepting a less than equal situation in which they live and living in a state of disempowerment. Resilience is what keeps them going.

Capabilities rely on both the assets available to the individual (human, social, educational and financial) and the social and political landscape that acts to enhance or constrain capabilities (Mitra 2006). Using the capabilities lens, structural violence served to eliminate the capabilities of families with disabled children, and ultimately the children themselves. The article has therefore brought out evidence-based challenges associated with disability in the context of wider development challenges.

## Conclusion

Politics resulted in uprooted and illiterate communities, diseases, disability, loss of dignity and death. This prevented the Tonga from meeting their basic needs, with structural violence appearing under names such as discrimination, exploitation and injustice. The Tonga situation needs government’s political will to eradicate social suffering through development programmes that will create employment, adequate social services, infrastructure, safeguard human rights and compensation for the forced displacement. The cerebral palsy incidence and disability can be minimised bringing relief to the informants and their disabled children. This will be in line with the two UN Conventions (CRC, UN 1989; CRPD, UN 2006) and the ‘African Charter on the Rights and Welfare of the Child’ (1990 – and entered into force in 1999) which urge member states including Zimbabwe to prioritise disability as social development policies and programmes by addressing structural violence.
